# The effects of different multidirectional plyometric sequences on shooting, balance, and neuromuscular performance in professional male basketball players

**DOI:** 10.1371/journal.pone.0331791

**Published:** 2025-09-12

**Authors:** Seifeddine Brini, Fatma Hilal Yagin, Julio Calleja-González, Pierpaolo Sansone, Georgian Badicu, Anissa Bouassida, Carlo Castagna, Gianpiero Greco, Abdullah F. Alghannam, Luca Paolo Ardigo, Anne Delextrat

**Affiliations:** 1 Research Unit, Sportive Performance and Physical Rehabilitation, High Institute of Sports and Physical Education of Kef, University of Jendouba, Kef, Tunisia; 2 Department of Biostatistics, Faculty of Medicine, Malatya Turgut Ozal University, Malatya, Turkey; 3 Department of Computer Science, Lakehead University, Thunder Bay, Canada; 4 Physical Education and Sport Department, Faculty of Education and Sport, University of the Basque Country (UPV/EHU), Vitoria, Spain; 5 Department of Education and Sports Sciences, Pegaso Telematic University, Naples, Italy; 6 Department of Physical Education and Special Motricity, University Transilvania of Brasov, Braşov, Romania; 7 School of Sports and Exercise Science, University of Rome Tor Vergata, Rome, Italy; 8 Technical Department, Fitness Training and Biomechanics Laboratory, Italian Football Federation (FIGC), Florence, Italy; 9 Department of Translational Biomedicine and Neuroscience (DiBraiN), University of Study of Bari, Bari, Italy; 10 Lifestyle and Health Research Center, Princess Nourah bint Abdulrahman University, Riyadh, Saudi Arabia; 11 Department of Teacher Education, NLA University College, Oslo, Norway; 12 Centre for Movement, Occupation and Rehabilitation Services, Oxford Brookes University, Oxford, United Kingdom; University of Split, CROATIA

## Abstract

**Objective:**

Repeated multidirectional jumps are highly specific movements in basketball, which may be important to integrate the training routine. Accordingly, this study aimed to assess the effects of three different multidirectional plyometric jumping sequences on shooting, balance, and neuromuscular performance in professional, male basketball players.

**Methods:**

Eighteen players underwent familiarization and baseline performance assessments in an initial session. Players then completed 21 visits in a randomized, counterbalanced manner. Each visit involving 10 repetitions of one of three different multidirectional plyometric protocols followed by a single performance test (countermovement jump (CMJ), squat jump (SJ), five jump (FJT), change-of-direction T-test (CoD T), Stork balance (SBT), Y-balance (YBT), three-point shooting (3pts) tests). The first plyometric protocol (P1) consisted of a combination of vertical and horizontal jumps, the second protocol (P2) consisted of a combination of drop, lateral, and vertical jumps, and the third protocol (P3) consisted of a combination of drop, single-leg step-up, and horizontal jumps. Each session was separated by at least 48 h of rest and recovery time between repetitions was 20 s.

**Results:**

Neuromuscular (CMJ, SJ, FJT and CoD T), body balance (SBT and YBT), and Shooting performances were significantly worse following each plyometric protocol compared to baseline (p < 0.001; d = 0.23–2.21). In addition, the majority of the measured performances were significantly lower following P3 compared to P1 and P2.

**Conclusions:**

Basketball-specific performance was adversely influenced by repeated multidirectional plyometric routines. Incorporating these particular strategies into the training regimen to counteract the induced tiredness will be intriguing.

## 1. Introduction

The introduction During basketball games, repeated multidirectional jump sequences are frequently performed concomitantly with other explosive actions such as sprints and shuffles as well as technical actions like jump shots and blocks [1]. Such jump sequences can be executed in vertical or horizontal directions, or a combination of both [2]. This discernment is important given the stretch-shortening cycle (SSC) contributes less in horizontal jumps than vertical jump given vertical loading of the musculo-tendinous unit ac-cumulates greater elastic energy during movement the eccentric phase [3]. Despite their mechanical differences, vertical and horizontal jumping have been shown to yield greater strength and balance improvements in team sport athletes than either vertical or horizontal jumping performed separately [4].

Additionally, some vertical and horizontal jump techniques employ stepping from a raised height immediately prior to the jump. This technique is known as a drop jump (DJ) and was extensively used to increase muscle performance in power-oriented activities such as jumping, throwing and sprinting especially in basketball [5]. Recently, these authors suggested that plyometric protocol based on DJ may signiﬁcantly improve repeated sprint ability (RSA), jumping, sprinting, and change of direction (CoD) performances in basketball players. Moreover, they suggested that DJ protocols emerge as a practical and efficient means to enhance high-intensity exercise performances in athletes. In the same context, Matavulj et al. [6] reported that a training program including a DJ protocol led to a better vertical jump and maximal voluntary force production in elite basketball players. Additionally, plyometric training with rapid SSC involving both vertical and horizontal movements may enhance body balance for players to better control their body position during dynamic tasks [7–9]. Although plyometric exercises have been widely used as training methods to improve the SSC efficiency [10,11], a need remains to examine their applicability in specific competitive conditions.

In this topic, several studies reported a significant relationship between jump shot, lower limb power, neuromuscular performance and stability among Basketball players [12–14]. Moreover, it is well known that Basketball players need to have a strong and stable lower limb in order to achieve some specific basketball actions such as jump shots which is a crucial factor of basketball performance, especially three-point shooting (3pts) efficiency which is highly associated to its final outcome [15]. This puts pressure on your knees and can throw you off balance. Additionally, it is important for basketball players to have a good balance especially during the landing phase to avoid injuries in lower limbs. Additionally, the identification and the subsequent management of fatigue may prevent detrimental physical and physiological adaptations often associated with injury and en-hance athletic performance and player availability [16]. In this context, the effect of fatigue induced by the plyometric sequences during games especially in basketball need to be well investigated and understandable for coaches in order to well manage training session and improve performances.

Otherwise, in order to have a good shot positions players undergo many repeated heigh efforts sequences (i.e multidirectional sprint/jump) in addition players may be tired under these conditions. Especially that the high intensity movement demands and physiological stress on the athletes during competition may accumulate over the pre-season and competitive season and present as signs of fatigue leading to decreased performance output and/or injury [17]. Thus, it will be interesting to investigate the effects of basket-ball-specific fatigue on 3PS accuracy.

To the best of our knowledge, few studies have examined the acute effects of multi-directional plyometric movement sequences on performance attributes in basketball players. Therefore, the objective of this study was to assess the effects of different repeated multidirectional plyometric sequences on shooting, balance, and neuromuscular performance in professional, male basketball players.

## 2. Materials and methods

### 2.1. Participants

Eighteen professional, male basketball players (age: 24.3 ± 2.2 years; body height: 1.88 ± 0.15 m; body mass: 76.5 ± 7.2 kg; maximal oxygen consumption (VO2max) 53.1 ± 2.3 ml ∙ min-1 ∙ kg-1) from the Tunisian first division basketball competition (training 4–5 days per week, 90 min per day, training experience 12.3 ± 3.5 years) volunteered for this study. An a priori power calculation showed a required minimum sample size of 12 players with β = 0.80, and α = 0.05 (G*Power v. 3.0). Players occupied 1 (point guard), 2 (shooting guard), and 3 (wing) playing positions. All players were eligible for inclusion in this study given they had no history of musculoskeletal, neurological, or orthopedic disorders that might have aﬀected their ability to perform the physical performance tests and plyometric jump protocols. The present study was approved by a local Clinical Research Ethics Committee (approval no. 12/2021). The experimental protocol was conducted according to the Declaration of Helsinki [18] and all players signed written informed consent after being made aware of the procedures, benefits, and risks before participating.

### 2.2. Design and procedures

**Fig 1:**
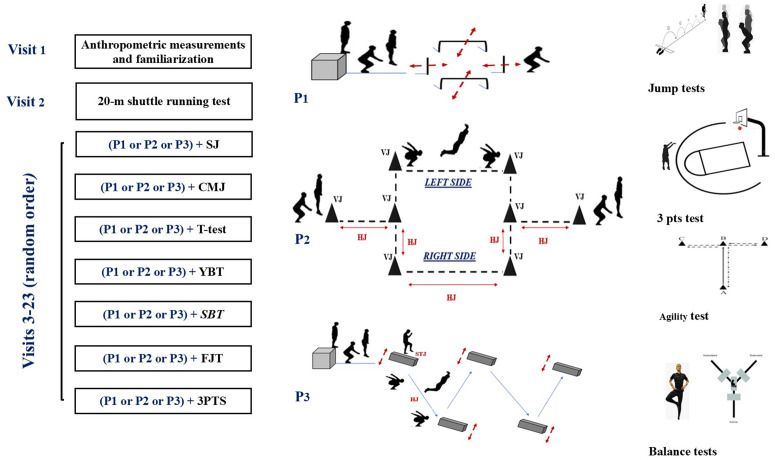
Experimental design. P1: First protocol; P2: Second protocol; P3: Third protocol; SBT: Stork balance test; YBT: Y-balance test; T-test: CoD T test; SJ: squad jump test, CMJ: countermovement jump test; FJT: five jump test.

The study was performed across a 6-week period at the end of the 2022–2023 competitive season. All-over the experimentation, players wore their typical basketball attire and arrived at the same gymnasium at 8:00 am for each testing session to minimize any effects of diurnal variations on physical performance [19]. Players had the same breakfast prior to each session (coffee with semi-skimmed milk, a healthy snack (Moulin d’Or, 60 g), and a yogurt) [20], after which no further food intake was permitted until testing was completed. All participants completed the study according to the previously described methodology. No injuries occurred over the course of the study. Adherence rates were 98.1%. Each player visited the laboratory on 23 occasions in total (Fig 1). The first visit entailed anthropometric assessment, baseline measurements for all performance attributes, and familiarization with the plyometric protocols. The second visit involved aerobic assessment via estimation of maximal oxygen consumption (VO2max) from the 20-m multistage shuttle run test as previously documented [21]. For the remaining 21 visits, players performed one of three different multidirectional plyometric jump protocols followed by the completion of a single performance test in a randomized, counterbalanced manner. each plyometric protocol consisted of 10 repetitions, with the first protocol (P1) involving a combination of vertical and horizontal jumps, the second protocol (P2) involving a combination of drop, lateral, and vertical jumps, and the third protocol (P3) involving a com-bination of drop, single-leg step-up, and horizontal jumps (Fig 1). One of seven different performance tests were completed immediately following the plyometric protocol in each session, including SJ, a CMJ a FJT, CoD test, a Stork static balance test, Y-balance test or a 3pts. Each session was separated by at least 48 h of rest, and recovery time between repetitions was 20 s. Plyometric protocols were performed on the same hardwood basketball court, adjacent to the three-point line to ensure last jump and the shots (similarly, the jumps were performed very close to the finish line of the sprints). Heart rate (HR) was continuously measured for each player through-out the plyometric protocol using (Polar Team Sport System; Polar-Electro OY, Kempele, Finland) to derive average and maximum values (beats·min-1). In turn, rating of perceived exertion (RPE) using a 1–10 modified scale [22] and blood lactate concentration (mmol·L-1) via fingertip capillary sampling were measured 3 min following test completion.

During the experimental period, the daily training regimen was conducted at a moderate intensity level, tailored to the athletes’ physiological and psychological state at the end of the competitive basketball season. Each session was systematically structured to include a general warm-up, targeted fundamental basketball skill drills, and moderate-intensity exercises emphasizing mid-range shooting and free throws. Additionally, sessions incorporated technical and tactical components designed to reinforce game-related decision-making and execution. This training structure aimed to maintain performance while minimizing fatigue and the risk of overtraining during the recovery-sensitive post-season phase.

### 2.3. Performance attributes

#### 2.3.1. Neuromuscular performance.

2.3.1.1. Vertical jump tests

Vertical jump height (cm) was evaluated using an optoelectrical system (Opto-Jump Microgate, Italy) using the following equation: jump height = 1/8 × g × t 2, where g is the acceleration (m·s-2) due to gravity and t (s) is the flight time [23]. Players performed the countermovement (CMJ) and the squat jumps (SJ) according to previously described protocols [24]. Three trials for each test were completed and the best trial was used for further analysis.

2.3.1.2. Five-time jump test (FJT)

The FJT consists of 5 consecutive strides with joined feet position at the start and end of the jumps. From the starting joined feet position, the participant was not allowed to perform any back step with any foot; rather, he had to directly jump to the front with a leg of his choice. After the first 4 strides, i.e., alternating left and right feet for 2 times each, he had to perform the last stride and end the test again with joined feet [25]. Performance was measured with a tape measure from the front edge of the player’s feet at the starting position to the rear edge of the feet at the final position [25]. FJT performance was recorded to the nearest 0.01 m. Three trials were completed and the best trial was used for further analysis.

2.3.1.3. Change-of-direction T-test (CoDT)

The CoDT is a valid test to evaluate change-of-direction speed in basketball players as it includes forward, lateral, and backward running over short distances [26]. Two trials were completed with 2 min of rest between trials and the fastest performance time taken for further analysis. Performance time was recorded to the nearest 0.01 s using an electronic timing system (Brower Timing Systems, Salt Lake City, UT, USA) placed 0.4 m above the ground.

#### 2.3.2. Balance performance.

2.3.2.1. Stork balance test (SBT)

The SBT was utilized to assess static balance [27,28]. Players stood on one foot, with their raised foot positioned against the inside of the supporting knee and both hands on the hips. On the “go” command, players raised the heel from the floor and held this position for as long as possible. The test was terminated when the heel of the supporting leg touched the ground, or the non-supported foot moved away from the knee. The test was timed (s) using a stopwatch. Three trials were performed interspaced by 2 min of rest with the longest trial used for further analysis.

2.3.2.2. Y-Balance Test (YBT)

The YBT has been described previously, with strong reliability [28]. Reach directions were evaluated by affixing three separate tape measures to the floor – one oriented anteriorly and the other two oriented at 135° in the posterior-medial and posterior-lateral directions. All testing was conducted barefoot where players stood on the dominant leg, with the most distal aspect of the hallux at the center of the grid. Players then reached in the specified direction, while maintaining a single-limb stance. Tests were classified as invalid if players (1) did not reach the line with their foot while maintaining a single-leg stance, (2) lifted the supported foot from the center grid, (3) lost balance at any point during the trial, (4) did not maintain start and return positions for 1 s, or (5) contacted the non-supported foot with the ground to gain support [29]. Players completed three test trials in each direction interspersed with 2 min of test. The best trial was used for further analysis.

#### 2.3.3. Shooting performance.

2.3.3.1. Three point shot test (3pts)

The 3pts has been used in a recent study [30] and consisted of 10 consecutive 3-point shots including two consecutive shots from the two corners, the two wings and the point guard position with an official basketball (Molten gf7, 600 g). The percentage of successful shots was taken for further analysis, which was calculated as: (number of shots made/ 10) x 100 [30]. Three trials were completed and the best trial was used for further analysis.

### 2.4. Statistical analyses

Statistical analyses were performed using SPSS version 24 for Windows (SPSS Inc, Chicago, Il, USA). Given the normality of all data was confirmed using the Shapiro-Wilk test, variables are presented as mean ± SD. One-way repeated-measures analyses of variance (ANOVA) with Bonferroni post-hoc tests were undertaken to assess differences performance attributes between baseline, P1, P2, and P3. Effect sizes for each ANOVA were calculated using partial eta squared (ηp2), while effect sizes for pairwise post-hoc comparisons were determined using Cohen’s d and interpreted as small (>0.10–0.30), medium (>0.30–0.50), and large (>0.50) [31]. 95% confidence intervals for the differences tested (95% CI) were also shown. The reliability of the tests was assessed by the Intraclass Correlation Coefficient (ICC) (Table 1). Statistical significance was accepted when p < 0.05 in all analyses.

**Table 1:** Intraclass correlation coefficients (ICCs) for relative reliability and coefficients of variation for absolute reliability of the applied physical fitness tests.

Measures			ICC	95%CI	%CV
**SJ (cm)**			0.98	0.91–0.98	3.5
**CMJ (cm)**			0.97	0.90–0.98	3.7
**FJT(m)**			0.98	0.88–0.97	3.3
**T_test (s)**			0.98	0.91–0.98	3.6
**STB (s)**	**R**		0.90	0.82–0.94	3.4
	**L**		0.89	0.83–0.93	3.2
**YBT (cm)**	**R**	***Ant***	0.97	0.85–0.97	5.1
		***Post/Md***	0.98	0.86–0.98	5.2
		***Post/Lat***	0.98	0.85–0.98	5.1
	**L**	***Ant***	0.97	0.84–0.98	5.3
		***Post/Md***	0.96	0.86–0.97	5.1
		***Post/Lat***	0.98	0.84–0.98	5.1

ICC – intraclass correlation coefficient; CI – confidence interval; CV – coefficient of variation (%); SBT: Stork balance test; YBT: Y-balance test; R: right leg; L: left leg; Ant: anterior; Post/Md: postero-medial; Post/Lat: postero-lateral; T-test: CoD T test; SJ: squad jump test, CMJ: countermovement jump test; FJT: five jump test.

## 3. Results

### Neuromuscular performance

ANOVA revealed a significant main effect between conditions for CMJ, SJ, FJT, and TCOD [(CMJ: p < 0.001; ES = 0.79; large); (SJ: p < 0.001; ES = 0.85; large); (FJT: p = 0.009; ES = 0.31; medium); (TCOD: p < 0.001; ES = 0.77; large); respectively] (Table 2). In turn, post-hoc analyses demonstrated neuromuscular performance following each plyometric protocol was significantly worse than baseline [(CMJ: p < 0.001, d = 0.28–0.40); (SJ: p < 0.001, d = 0.32–0.34); (TCOD: p < 0.001, d = 0.006–0.01)]. Moreover, performances were significantly poorer after P3 than following P1 and P2.

**Table 2:** Variation of body balance, change-of-directions and Jump performances following the three different multidirectional plyometric protocols.

Variables			BASLINE	P1	P2	P3	*p*-value (effect size)
**SJ (cm)**			42.39 ± 1.46	39 ± 1.33	39.66 ± 1.14	37.05 ± 0.94	< 0.001(0.85)
**CMJ (cm)**			43.33 ± 4.42	40.83 ± 4.58	40.22 ± 3.84	38.72 ± 4.55	< 0.001(0.79)
**FJT(m)**			8.20 ± 0.71	8.07 ± 0.42	8.10 ± 0.44	7.98 ± 0.41	0.009 (0.31)
**T_test (s)**			6.58 ± 0.07	6.63 ± 0.06	6.64 ± 0.07	6.70 ± 0.07	< 0.001(0.77)
**STB (s)**	**R**		22.15 ± 1.74	20.76 ± 1.15	20.88 ± 1.41	19.49 ± 1.01	< 0.001(0.66)
	**L**		21.95 ± 1.70	20.32 ± 1.36	20.64 ± 1.38	19.24 ± 1.24	< 0.001(0.69)
**YBT (cm)**	**R**	***Ant***	91.72 ± 4.91	88.61 ± 2.85	86.06 ± 2.80	84.72 ± 3.41	< 0.001(0.77)
		***Post/Md***	102.22 ± 4.49	96.55 ± 4.22	93.45 ± 2.85	94.11 ± 4.47	< 0.001(0.73)
		***Post/Lat***	63.39 ± 2.28	60.78 ± 2.34	62.05 ± 2.10	59.06 ± 2.21	< 0.001(0.83)
	**L**	***Ant***	93.38 ± 2.75	88.33 ± 3.02	89.06 ± 2.88	86.81 ± 3.96	< 0.001(0.72)
		***Post/Md***	104.17 ± 3.96	99.61 ± 2.15	99 ± 2.11	97.56 ± 2.06	< 0.001(0.68)
		***Post/Lat***	62.11 ± 2.11	58.56 ± 1.20	57.39 ± 1.75	55.78 ± 1.22	< 0.001(0.80)

Data are reported as means and standard deviations. P1: First protocol; P2: Second protocol; P3: Third protocol; SBT: Stork balance test; YBT: Y-balance test; R: right leg; L: left leg; Ant: anterior; Post/Md: postero-medial; Post/Lat: postero-lateral; T-test: CoD T test; SJ: squad jump test, CMJ: countermovement jump test; FJT: five jump test.

### Balance performance

ANOVA revealed a significant main effect between conditions for STB [(Right: p < 0.001; ES = 0.66; large); (Left: p < 0.001; ES = 0.69; large); respectively)] (Table 2). In turn, post-hoc analyses demonstrated STB following each plyometric protocol was significantly worse than baseline [(Right: p < 0.001, d = 0.23–0.37); [(Left: p < 0.001, d = 0.21–0.35)]. Moreover, performances were significantly lower after P3 than following P1 and P2. Significant main effects were shown between conditions for YBT [Right (Ant: p < 0.001; ES = 0.77, large); (Post/Md: p < 0.001; ES = 0.73; large); (Post/Lat: p < 0.001; ES = 0.83; large); Left (Ant: p < 0.001; ES = 0.72; large); (Post/Md: p < 0.001; ES = 0.68; large); (Post/Lat: p < 0.001; ES = 0.80; large); respectively) (Table 2). Post-hoc analyses demonstrated YTB following each plyometric protocol was significantly worse than baseline [Right (Ant: p < 0.001; d = 0.68–0.78); (Post/Md: p < 0.001; d = 0.85–0.99); (Post/Lat: p < 0.001; d = 0.29–0.34); Left (Ant: p < 0.001; d = 0.60–0.83); (Post/Md: p < 0.001; d = 0.87–0.92); (Post/Lat: p < 0.001; d = 0.54–0.61)]. Moreover, performances were significantly lower after P3 than following P1 and P2.

### Shooting performance

**Fig 2:**
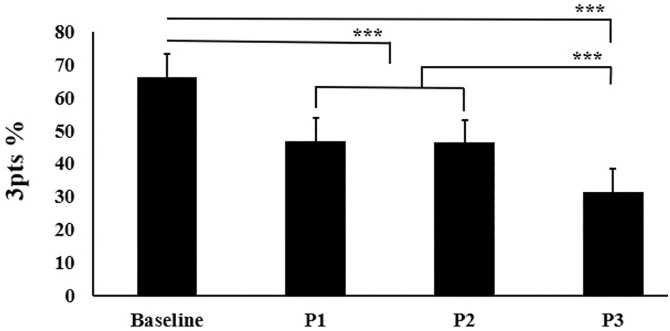
Performance in the three-point shot at baseline and immediately following the three different multidirectional jump protocols. ***p < 0.001. P1: First protocol; P2: Second protocol; P3: Third protocol.

ANOVA revealed a significant main effect between conditions for shooting performance (p < 0.001; ES = 0.83; large). In turn, post-hoc analyses demonstrated shooting performance following each plyometric protocol was significantly worse than baseline (p < 0.001, d = 1.45–2.21) (Fig 2). Moreover, shooting performance following were significantly poor-er after P3 than following P1 and P2.

## 4. Discussion

Authors The objective of this study was to assess the effects of different repeated specific multidirectional plyometric sequences on jump shots accuracy, body balance and neuromuscular performance in professional basketball male players. In general, our findings showed that the measured basketball specific performance such as vertical jump performances, lower limbs power, CoD performance, and body balance were negatively affected immediately following the three different protocols in compared to the baseline performances and with a significant poorer performance recorded after performing P3. Concerning, RPE values and HRavg, our findings didn’t register any significant differences following the different protocols. Moreover, most of the tests used in the present experimentation showed a good test-retest reliability in terms of ICC (>0.80). These results were similar to previous studies in the basketball field [2,20,32,33].

To our knowledge, our study is the first to examine jump performance and lower limb power following different repeated specific multidirectional plyometric sequences, which makes the comparison with the literature difficult for this condition. Our results post the three specific plyometric protocols were negatively affected with a lower value post P3. Our results are in accordance with the study of Esformes et al. [34] semiprofessional male rugby players. This decrease in jump performance was explained by different causes such as the testing procedures, the rest period (5 min) between plyometric exercises, the number of repetitions and the duration of the plyometric intervention may have resulted in excess fatigue and decreased power capacity that probably suppressed any potentiating effects [35]. In the same context, Tillin and Bishop, [36] reported that per-forming more sets and/or repetitions of a conditioning activity, such as the tuck jumps, may induce fatigue.

Recently, Turki et al. [37], demonstrated that 3 sets of 3 tuck jumps were not adequate stimuli to increase the CMJ. Similarly, Tsolakis et al. [38], reported that 3 sets of 5 tuck jumps had no effect on CMJ height. Concerning the lower value in favor of P3, which is characterized by combination between drop jump, step up jump and horizontal jump, our findings were different from these who reported that DJ protocols have been extensively used to increase muscle performance in power-oriented activities such as jumping, throwing and sprinting [39–41]. Otherwise, there are few studies specifically evaluating the effects of DJ protocols in team sports for performance enhancement and more investigations need to be conducted in the future.

The FJT is a good alternative to vertical jumps when testing team sport players in the field, with no particular equipment required, hence our choice of the test in the present study. Within this context, our results suggest that the explosive power of the lower limbs during unilateral jumps (required for a lay-up for example) was negatively affected especially following P3 which is probably due to fatigue imposed by the nature of the repeated movements realized during this specific protocol especially that the step-up jump causes unilateral fatigue, as during this protocol participants perform several movements that require repeated force production from one limb. Thus, P3 used may be better served as training tools given their short-term impact on performance, but this remains to be investigated in a longitudinal training study for confirmation.

During the present investigation, the CoD speed which represent a decisive movement in basketball was negatively affected by the three protocols with a poorer value recorded following P3. To our knowledge, a direct comparison of our results to similar studies is hampered because research is often conducted with different experimental circumstances (Objectives, repetitions, movement’s nature/combinations) however, the only study conducted among basketball players that we can compare our results with is that of [5] who investigated the effects of drop jumps on repeated sprint ability with CoD among professional basketball players, and found a greater best time, total time, and mean time after the drop jumps condition which was in contradiction with our findings. Those authors attributed this improvement in performance to a potential PAP effect. However, these results could not be comparable to our findings because of the differences in the protocols in term of repetitions, combinations and recovery times.

A recent systematic review of the effects of PAP on sprint and CoD performance in athletes from various sports indicated that just one research found significant evidence that such regimens may increase sprint performance but little evidence for improvements in CoD ability [42]. Aloui et al. [43] found that 34 male football players improved their sprint performance and CoD ability following eight weeks of exposure to a PAP warm-up program. However, these results should not be compared to our investigation because of probable chronic adaptations, which might explain the improvements in contrast to our findings. Otherwise, the decrease in CoD performance observed in the current study could be attributed to a variety of factors, including the volume, intensity, and type of the conditioning activity [44], as well as the recovery period between the conditioning activity and performance [36].

Shooting technique constitutes one of the most crucial abilities in basketball, and its precision may be the key factor for winning and losing a competition. In the same context, vertical jump is also considered one of the fundamental movement abilities used in basketball shooting [12]. According to Rodríguez et al. [13], the maximal jump height is a re-liable predictor of leg muscle strength and a key factor in basketball performance, including rebounding, blocking, and shooting. In addition, many specific movements such as vertical jump and balance are a prominent element for movement quality such as jump shooting [45]. Furthermore, it is suggested that balance help athletes to stay in the air throughout jumping and has a significant impact on their performance [14]. In this con-text, the finding of the present study showed a significant poorer body balance and 3pts shooting percentage following the three protocols with worst value reported following P3.

The main explanation for the results obtained is based on the deterioration of jump performance and lower limb strength due to fatigue accumulated after the different proto-cols which may affect jump shot and balance, more specifically the third protocol which consisted of the combination of drop jump, single leg step-up jump and horizontal jump especially that it was reported that there were statistically significant correlations between static balance and drop jump in athletes [46]. Thus, it is recommended that more drop jump exercise should be allocated in athletes’ training programs for the development of static balance performance and for the reduction of sports injuries. Furthermore, when the relevant literature is examined, there are research findings showing that vertical jump performance positively affects shooting performance in basketball players [47,48].

### Limitations

Although we present a novel addition to the literature, our study has some limitations that warrant consideration. First, the specific sequences were chosen with certain combinations of jumps – however, different results may be elucidated if different types of jumps, repetitions, and intensities are introduced Second, jumping sequences are not per-formed only without other specific basketball movements. Therefore, further studies should combine repeated jump, sprints or shuffling sequences using these movement patterns and fundamentals skills, with or without the ball and in more directions to better replicate the reality of a basketball game. Also, separate performance tests were used whereas actual in-game performance would provide greater ecological validity. Further-more, baseline was gathered at the end of the season, so changes may have been due to detraining – this is a major problem with the design that I raised earlier. The players were also mostly backcourt players, so it is not really representative of all positions within a team.

## 5. Conclusions

The current study found that the three procedures had a detrimental effect on basketball-specific abilities, with poorer performance observed after P3. Coaches and physical trainers ought to incorporate and mimic these unique basketball circumstances while training these talents in order to optimize training responses and improve performance by to counteract the induced fatigue.

### Practical applications

- Repeated lateral single leg step up should be included into the training routine of basketball players in order to improve some specific basketball movement.
- Combing different models of plyometric training may improve specific basketball performances to better replicate the reality of a basketball game.
- Three points jumps shots are an important key factor especially in the money time, however, the fatigue imposed at the end of games may influence these important weapons. Thus, some related physical abilities such as vertical jumping and balance need to incorporated and repeated during training sessions in order to improve it.

## Supporting information

S1 The excel Data includes the participants performances (n = 18) recorded during the present investigation (The Static Balance test, the Y-Balance test, the 3pts shot test; the T-test, the Squad jump test, the counter jump test, and the five jump test; respectively). (XLSX)

## List of abbreviations:

SBT: the Stork test
YBT: Y-Balance Test
T-test: CoD test
HR: heart rate
RPE: The rating of perceived exertion
SJ: the squat jump
CMJ: countermovement jump
FJT: the five time-jump test
CoD: change of direction
P1: First protocol
P2: Second protocol
P3: Third protocol
